# Traditional Uses of Cannabinoids and New Perspectives in the Treatment of Multiple Sclerosis

**DOI:** 10.3390/medicines5030091

**Published:** 2018-08-15

**Authors:** Francesca Gado, Maria Digiacomo, Marco Macchia, Simone Bertini, Clementina Manera

**Affiliations:** Department of Pharmacy, University of Pisa, Pisa 56126, Italy; francesca.gado@for.unipi.it (F.G.); maria.digiacomo@unipi.it (M.D.); marco.macchia@unipi.it (M.M.)

**Keywords:** multiple sclerosis, endocannabinoid system, cannabinoid receptors, monoacylglycerol lipase (MAGL) inhibitors, fatty acid amide hydrolase (FAAH) inhibitors, arachidonoylethanolamine (AEA) reuptake inhibitors

## Abstract

Recent findings highlight the emerging role of the endocannabinoid system in the control of symptoms and disease progression in multiple sclerosis (MS). MS is a chronic, immune-mediated, demyelinating disorder of the central nervous system with no cure so far. It is widely reported in the literature that cannabinoids might be used to control MS symptoms and that they also might exert neuroprotective effects and slow down disease progression. This review aims to give an overview of the principal cannabinoids (synthetic and endogenous) used for the symptomatic amelioration of MS and their beneficial outcomes, providing new potentially possible perspectives for the treatment of this disease.

## 1. Introduction

Multiple sclerosis (MS) is an important neurological disease that affects the central nervous system (CNS). It is the most common neurological disorder in young adults and affects approximately 2.3 million people worldwide [1]. It is more common in women than in men [2,3], with a prevalence ratio of 3:1 [4,5]. Regarding its etiology, it is now widely accepted that genetic and environmental factors may contribute to the onset and development of the disease [6,7]. MS is a chronic inflammatory immune-mediated condition characterized by demyelination of the axons in the CNS. It gradually leads to progressive neurodegeneration that damages CNS myelin, leading to neuronal dysfunction and a broad spectrum of neurological symptoms that depend upon the site where lesions have occurred in the brain and spinal cord. The symptoms of MS include spasticity, sensory alterations, weakness, painful spasms, bladder dysfunction, tremor, ataxia, optic neuritis, fatigue, and dysphagia [8].

On the basis of the understanding of the disease process in MS and of magnetic resonance imaging (MRI) technology, in 1996, the International Advisory Committee on Clinical Trials of MS, classified MS into four independent subtypes [9]: (1) relapsing-remitting MS (RRMS), which is the most common form of MS (approximately 85%–90% of all cases) [8] and it is typified by unpredictable relapses with full recovery or with sequelae; (2) secondary progressive MS (SPMS), which follows generally RRMS (approximately 65% of RRMS patients will evolve to SPMS), progressing and leading to neurological damages and consequently physical decline of the patient without remission [10]; (3) primary-progressive MS (PPMS) which is diagnosed in approximately 10%–15% of patients [8] and progresses continuously from the onset without attacks; and (4) progressive-relapsing MS (PRMS) which is the least common subtype of MS, includes approximately 5% of all cases of MS and characterized by a steady decline onset with super-imposed attacks. However, in 2013, the Committee revised the classification, retaining the basics of 1996 MS phenotypes description, with some modifications and clarifications. These include the elimination of the PRMS category, since subjects so categorized would be classified as PP patients (PP-active if acute attacks are present, PP-inactive if no acute attacks occur), and the inclusion of the clinically isolated syndrome (CIS) recognized as the first clinical presentation of a disease that shows characteristics of inflammatory demyelination that, if subsequently active and fulfilling MS diagnostic criteria, becomes RRMS [11].

Molecular mechanisms of MS progression remain unclear. However, the observed hallmarks are considered as consequence of three synergistically mechanisms: inflammation, demyelination, and axonal damage. Recent evidence indicates that MS is primarily a neurodegenerative disease that starts in the brain and then develops because of inflammation [12]. This hypothesis has led to two models of MS immune-pathogenesis: the “inside-out” and “outside-in.” In the first model, a dysfunction of brain cells causes the immune response that destroys myelin and leads to blood-brain barrier (BBB) breakdown. In the second model, a dysfunction of the periphery leads to BBB damage, myelin disruption, and axonal death [13]. The subsequent high presence of lymphocytes in the CNS and the activation of innate immune cells (dendritic cell, macrophages, microglia) play key roles in MS pathogenesis. The activation of autoimmune cells, resident microglia, astrocytes and macrophages, results in an immunological storm that involves abundant secretion of reactive species, cytokines, chemokines, autoantibody production, and enhanced excitotoxicity. There is a continuing activation of resident microglia and astrocytes producing pro-inflammatory mediators that potentiate the neuroinflammatory response. This results in oligodendrocytes and axonal damage and ultimately in demyelination, synaptic alteration, and neuronal loss [8,14,15,16]. In the early phases of MS, the oligodendrocytes generate new myelin, and this remyelination is one of the reasons why symptoms decrease or temporarily disappear in RRMS [17]. However, the myelin sheaths are not completely rebuilt by oligodendrocytes, and repeated attacks lead to damage in the axons where scar-like plaques build up with subsequent axonal loss [18], associated with the characteristic symptoms of MS [19].

At present, there are three categories of available drug therapies for MS: treatment of clinical attacks (acute relapses), disease-modifying, and symptomatic therapies.

The first-line treatment of acute relapses in MS consists of intravenous administration of high doses of corticosteroids, specifically methylprednisolone (MP) [20]. For patients who do not improve or cannot tolerate MP, there is a second line therapeutic option that is represented by adrenocorticotropic hormone (ACTH) in a gel formulation administered either intramuscularly or subcutaneously [20,21].

Disease-modifying therapies (DMTs) are aimed to reduce the relapses in terms of number, duration, and gravity while maintaining remission and slowing progression. These therapies also reduce disability, MRI lesions, and should improve the quality of life of MS patients. First-generation injectable DMTs that are currently approved by the Food and Drug Administration (FDA) include four interferon beta formulations and glatiramer acetate. Four orals (fingolimod, teriflunomide, dimethyl fumarate, and cladribine) and three monoclonal antibodies (natalizumab, alemtuzumab, and ocrelizumab, all intravenous injectables) are also indicated for MS treatment [22,23,24,25,26].

Interferon beta, glatiramer acetate, teriflunomide, and dimethyl fumarate are considered first-line therapies, while natalizumab and alemtuzumab are second-line drugs. In the European Union, fingolimod is approved as a second-line treatment, while in the United States, Canada and other countries it is a first-line drug [27].

Symptomatic therapies that relieve the distressing and/or disabling symptoms of multiple sclerosis include muscle relaxants for spasticity (baclofen, tizanidine, dantrolene sodium, benzodiazepines), anticonvulsants for neuropathic pain (diazepam, clonazepam, gabapentin, pregabalin) [28,29], and anticholinergic drugs for bladder dysfunction and dysphagia (oxybutynin, propantheline) [8,30,31]. However, limitations of these therapeutic options result from their incomplete effectiveness in managing such complex symptoms and adverse reactions associated with their prolonged use [32,33]. A therapeutic option for patients with severe refractory spasticity is the intrathecal delivery of baclofen via an implanted pump, although the procedure is invasive, expensive, and not free of possible complications (pump dysfunction, infection). Moreover, intramuscular botulinum toxin injections have been shown to be effective in the case of focal spasticity [33,34]. Finally, a temporary reduction in spasticity can be obtained by blocking peripheral nerves with phenol injections [35].

Over the last 15 years, a great amount of preclinical studies has demonstrated that compounds targeting the endocannabinoid system (ECS) exert anti-inflammatory properties and neuroprotective and immuno-modulatory effects [36], allowing them to alleviate symptoms and to limit progressive neurodegeneration in animal models of MS [37].

The endocannabinoid system consists of two G protein-coupled receptors, the type-1 (CB1R) and type-2 (CB2R) cannabinoid receptor, a class of lipids mediators called endocannabinoids (ECs), and several enzymes involved in the biosynthesis and degradation of ECs. The most well-studied ECs are N-arachidonoylethanolamine (anandamide, AEA) and 2-arachidonoylglycerol (2-AG) [38]. The biological activities of these lipid mediators are terminated upon cellular re-uptake and subsequent metabolism. The main metabolic enzymes of ECs are fatty acid amide hydrolase (FAAH) that degrades AEA into arachidonic acid (AA) and ethanolamine [39] and monoacylglycerol lipase (MAGL) that degrades 2-AG in AA and glycerol [40].

The aim of this review is to highlight the role of ECS in MS. In the first part, it will concern medicinal cannabinoids that are licensed for MS treatment. Subsequently, the main classes of ECS modulators described in the literature will be reviewed, focusing on their mode of action and on obtained results in preclinical studies that highlight their potential translational aspects for MS.

## 2. Medicinal Cannabinoids

Scientific research concerning the use of cannabinoids to manage MS symptoms has been stimulated by anecdotal reports of MS patients that experience symptomatic relief after smoking cannabis [41]. These evidence have produced a considerable interest for taking cannabinoid-based medicines into clinical practice for the treatment of both relapsing-remitting and progressive forms of MS. Therefore, various cannabinoid preparations have been examined in several clinical studies to assess their efficacy in symptomatic treatment of MS and other neurological disorders in human patients [42]. The medicinal grade cannabinoids that are licensed for MS treatment change from country to country. Off-label use also varies widely [42,43,44].

Currently, the three main licensed cannabinoid preparations are Marinol^®^ (whose active principle is dronabinol), Cesamet^®^ (whose active principle is nabilone, Table 1), and Sativex^®^ (whose active principle is nabixiomls, i.e., a standardized~1:1 (*w/w*) mix of ∆^9^-tetrahydrocannabinol (∆^9^-THC) (Table 1) and cannabidiol (CBD), both extracted from *Cannabis sativa*) (Table 1). There are also other preparations based on natural cannabinoids containing different quantitative ratios of of ∆^9^-THC and CBD, i.e., Bedrocan^®^ (22% ∆^9^-THC, <1% CBD from *Cannabis sativa*), Bedrobinol^®^ (13.5% ∆^9^-THC, <1% CBD from *Cannabis sativa*), Bediol^®^ (6.5% ∆^9^-THC, 8% CBD from *Cannabis sativa*), Bedica^®^ (14% ∆^9^-THC, <1% CBD from *Cannabis indica*), Bedrolite^®^ (<1% ∆^9^-THC, 9% CBD from *Cannabis sativa*), Bedropuur^®^ (20%–24% ∆^9^-THC, <1% CBD from *Cannabis indica*).

### 2.1. Dronabinol

“Dronabinol” is the generic official name for the synthetically obtained pure isomer of THC, (−)-*trans*-Δ⁹-tetrahydrocannabinol, the main THC isomer found in the cannabis plant. It was originally developed for the treatment of chemotherapy-induced nausea and vomiting (CINV). Its use was then extended to anorexia associated to weight loss in AIDS patients.

It is available in oral soft gelatin capsules with a range of three dosage (2.5, 5, and 10 mg). Dronabinol has been specifically evaluated for its effectiveness and safety in treating MS symptoms in 10 clinical studies published between 1981 and 2013 [45,46,47,48,49,50,51,52,53,54]; almost all of these studies are reported in 10 reviews published between 2003 and 2016 [33,69,70,71,72,73,74,75,76,77] that have been included in a systematic review of reviews on the basis of eligibility criteria of methodological quality (AMSTAR Tool: A MeaSurement Tool to Assess systematic Reviews) [78]. Eight key clinical outcomes in MS have been considered to provide an overview of the current findings about dronabinol, i.e. disability/disease progression, pain, spasticity, bladder function, ataxia/tremor, sleep, quality of life, and adverse effects [78]. A summary of clinical evidence for dronabinol is shown in Table 1.

Positive effects have been found mainly with regard to pain associated with MS [46,49,50,51]. Substantially, no change was noted in the assessment of ataxia and tremor [45,46,47]. Also regarding to disability and disease progression, there are no evident changes [45,46,47,48,54] (noteworthy is the recent CUPID study [54], which has provided a large amount of data regarding treatment with dronabinol, showing that it has no overall effect on the progression of MS). Indeed, in some cases, negative effects have been detected [45]. Regarding the quality of sleep, there are mixed findings, although they are mostly positive [46,50]. There are mixed findings also for the rest of the clinical outcomes (spasticity [45,46,50,51,52], bladder function [46,53], and quality of life [45,46]). Adverse effects to dronabinol have been described more frequently as mild to moderate; principally, they were dizziness, euphoria, dry mouth, fatigue, and drowsiness [45,46,47,49,50,51,52,53]. Cognitive impairment did not appear to be relevant, except in patients with pre-existing cognitive disfunctions [45,46,49,50,53]. Taken together, these results indicate that for dronabinol, the only significant clinical evidence relates to its ability to relieve pain associated with MS, while for the rest, it can be concluded that evidence are quite inconsistent.

### 2.2. Nabilone

Nabilone is a synthetic analogue of ∆^9^-THC bearing a dibenzopyran-9-one structure (Table 1). It is a racemic mixture consisting of (S,S)-(+)- and (R,R)-(−)-isomers and was originally licensed in 1985 for the treatment of CINV in patients who failed to respond adequately to conventional antiemetic treatments. The development of serotonin 5-HT_3_ receptor antagonists has partially supplanted the use of nabilone for this therapeutic application.

In more recent years, some evidence has emerged about the use of nabilone for the treatment of pain of various origin (e.g., neuropathic, chronic, cancer pain) and spasticity related to MS. However, over the last two decades, there has been a minimal amount of research on its use beyond its license. Nabilone is available as capsules in strengths of 0.25, 0.5, and 1 mg. It has been specifically evaluated for its effectiveness and safety in treating MS symptoms in three clinical studies. These has been published between 1995 and 2015 [55,56,57] and are reported in four reviews published between 2006 and 2015 [69,70,72,73] included in a systematic review of reviews on the basis of eligibility criteria of methodological quality (AMSTAR Tool) [78]. As mentioned above, the eight MS clinical outcomes considered were: disability/disease progression, pain, spasticity, bladder function, ataxia/tremor, sleep, quality of life, and adverse effects [78]. A summary of clinical evidence of nabilone is showed in Table 1.

Although the clinical trials are not abundant and the number of patients evaluated has been rather low, positive effects due to nabilone were found regarding pain [55], spasticity [55,56], and bladder dysfunction [55] associated to MS. Mixed findings, although mostly positive, resulted in the evaluation of quality of life [55,57]. In fact, one study found a significant improvement in objective rating of general health status [55]; in another study (in which nabilone was evaluated as an adjunctive drug to gabapentin) was found an improvement in patient global impression of change, but no statistically significant difference in “VAS_impact_” (impact of pain on patient’s daily activities, recorded using a visual analog scale) was found between nabilone and placebo group [57]. No studies were conducted about the effect of nabilone on sleep quality of MS patients and on disability/disease progression. With regard to adverse reactions to nabilone, moderate sedation, dizziness, and moderate weakness in the legs have been reported [55,56]. These data indicate that, for nabilone, there are specific positive conclusions for three clinical outcomes related to MS, i.e. pain, spasticity and bladder problems.

### 2.3. Nabiximols

Nabiximols is a specific extract from cloned plants of *Cannabis sativa*, first approved in Canada in 2005 for the treatment of cancer pain and neuropathic pain in patients affected by MS. In the following years, the drug was authorized in several European countries, and today, it is available in about 20 countries worldwide for the treatment of MS-related moderate to severe spasticity in patients who have not responded adequately to other anti-spasticity therapies.

It consists of an approximate 1:1 fixed ratio of ∆^9^-THC and CBD [82]. It was developed in response to widespread anecdotal reports that cannabis was a useful medicine for treating a number of MS-related symptoms. CBD, non-psychoactive isolated plant cannabinoid, was included in the drug essentially to ameliorate side effects due to ∆^9^-THC.

Nabiximols is the active principle of the trade drug Sativex^®^, a pharmaceutical product standardized in composition, formulation, and dosage. It is formulated as an oro-mucosal spray, containing 27 mg of ∆^9^-THC and 25 mg of CBD/1.0 mL, in an aromatized water-ethanol solution. Sativex^®^ is available as 5.5 mL spray bottles (which deliver up to 48 sprays) or as 10 mL spray bottles (which deliver up to 90 sprays). Each dose (spray or “puff”) delivers 0.1 mL of solution (2.7 mg of ∆^9^-THC and 2.5 mg of CBD). The oro-mucosal administration allows a slower absorption with respect to inhalation, avoiding the high plasma levels that occur when cannabis is smoked or vaporized. On the other hand, this route is more rapid and consistent than the oral administration [83], allowing rapid and direct access to the circulation through the mucosa with a faster plateau of plasma concentration and avoiding the problems of the oral route [79]. Moreover, the oro-mucosal formulation provides a simple delivery system, allowing the patient to self-manage a convenient and accurate titration of dosage.

Nabiximols has been widely evaluated for its effectiveness and safety in treating MS symptoms in 11 clinical studies published between 2004 and 2014 [58,59,60,61,62,63,64,65,66,67,68]; these studies are reported in 11 reviews published between 2006 and 2017 [69,70,72,73,74,75,76,77,79,80,81]. Most of these reviews have been included in a systematic review of reviews on the basis of eligibility criteria of methodological quality (AMSTAR Tool) [78]. The eight main clinical outcomes in MS that have been included to provide an overview of the current findings about nabiximols, are the same seen in the above paragraphs, i.e., disability/disease progression, pain, spasticity, bladder function, ataxia/tremor, sleep, quality of life, and adverse effects [78]. A summary of clinical evidence of nabiximols is shown in Table 1.

Regarding MS-related pain, most evidence supports the use of nabiximols for this pathological condition. In fact, in many randomized controlled trials (RCT) a significant reduction in numeric rating scales (NRS) and visual analogic scales (VAS) score was highlighted in the treated group respect to placebo group [58,59,60,61,62,63,64,65]. Some clinical evidence indicates that nabiximols is probably effective in the treatment of spasticity associated with MS [58,59,66,67]. These data mainly concern the patient’s subjective evaluation scales (NRS); in the objective evaluation scales, such as Ashworth scale (AS) and modified Ashworth scale (MAS), the results are in favor of nabiximols but, in some cases, not statistically significant [59,66]. However, in some studies, no change in spasticity was found [60,63]. There is some evidence that nabiximols is probably effective in reducing the number of bladder voids per day [68], but contradictory results emerged about its efficacy in reducing overall bladder symptoms related to MS [58,68]. Clinical studies carried out to date failed to demonstrate that nabiximols can produce any significant positive change in MS-induced tremor and ataxia [58,59], while statistically significant subjective improvement in sleep quality have been highlighted [58]. Regarding the quality of life, there are in general mixed findings, although in some cases the average number of MS patients treated with nabiximols who reported an improvement of the global impression of change was significant [58,61,65,66]. On the basis of extensive clinical evidence, the adverse effects associated with nabiximols treatment are referred as mild to moderate, and generally well tolerated. They include drowsiness, dizziness, headache, fatigue, impaired balance and disturbance in attention [58,59,60,61,62,63,64,66]. Regarding disability and disease progression, clinical studies evidenced no significant changes in some parameters, such as Barthel index of activity of daily living (ADL) and walking time (10 mt) [58,59].

Overall, these data indicate that nabiximols may represent a valid therapeutic options for pain, spasticity, and quality of sleep in MS patients, with low incidence of adverse effects that are in general not serious and well tolerated.

## 3. Endocannabinoid System Modulators

### 3.1. CB1R and CB2R Ligands

Cannabinoids exert neuroprotective effects acting at multiple molecular sites that are in all key cellular elements for the control of neuronal survival (e.g., neurons, astrocytes, resting and reactive microglia, oligodendrocytes) and also in key brain structures (e.g., BBB) [84]. These effects are due to activation of CB1R and CB2R.

CB1R is widely expressed within the CNS (cortical neurons and interneurons, oligodendrocytes, astrocytes) and also in several leukocytes infiltrating the brain [85]. Initially CB2R has been restricted exclusively to immune cells (macrophages, mast cells, B and T lymphocytes) and immune organs (spleen, thymus, lymph nodes) [86]. However, some evidence showed the expression of CB2R in microglia of the CNS [87], and more recently, it has been also reported to be expressed in brainstem neurons and astrocytes upon cellular activation by an insult or inflammation [36,88,89].

The multiplicity of action of cannabinoids allows to reduce the excitotoxicity by acting through neuronal CB1R, as well as the toxic influence of reactive microgliosis by acting through microglial CB2R, or enhancing the trophic and metabolic support to neurons by acting through astroglial CB1R or CB2R. In particular, the activation of CB1R provides neuroprotection regulating glutamate homeostasis [90]. In fact, it is well-known that glutamate is a key mediator in neuronal and oligodendrocyte damage in MS [91], and CB1R agonists exert direct neuroprotective effects by limiting glutamate release and the excitotoxic damage characteristic of several neurodegenerative disorders [92]. Furthermore, the protective effects of CB2R activation in microglial cells upon inflammatory-induced CNS damage have been demonstrated in preclinical models of multiple sclerosis [92]. Microglia may be, in two activated states: M1 and M2. The classical M1 state is characterized by release of pro-inflammatory factors, i.e., interleukins (IL-1beta, IL-18, and IL-6), prostanoids and inducible nitric oxide synthase (NOS2)-derived NO. On the other hand, the neuroprotective M2 state, known as “alternative activation” is associated with the release of anti-inflammatory factors, such as IL-10, IL-4, and NGF [93]. Microglia has a functional endocannabinoid signaling system, composed of cannabinoid receptors and the complete machinery for the synthesis and degradation of endocannabinoids. The expression of cannabinoid receptors, mainly CB2R, and the production of endocannabinoids have been related to the activation profile of these cells [94].

In preclinical studies, the beneficial effects of cannabinoids have been reported in different animal models of MS including experimental autoimmune encephalomyelitis (EAE), chronic relapsing experimental allergic encephalomyelitis (CREAE), and Theiler’s murine encephalomyelitis virus induced demyelinating disease (TMEV-IDD) [95]. More specifically, autoimmune encephalomyelitis is a demyelinating autoimmune disease of the CNS that is characterized by mononuclear cell infiltration and mainly induced by auto-reactive CD4^+^ T cells. EAE is a useful animal model of MS since many of the pathologies observed in the CNS of mice with EAE show strong similarity to those found in the CNS of MS patients [96]. CREAE animal model also presents relapsing-remitting paralytic episodes and tremor and spasticity of limb muscles during post-relapse remission strongly similar to MS [97]. Finally, TMEV-IDD is an immune-mediated demyelinating disease dependent on persistent virus infection of the macrophages, microglia and astrocytes within the CNS [98] and the inflammation and demyelination observed in TMEV-IDD are similar to those described in MS patients [99].

One of the first studies of cannabinoids in MS model is reported by Lyman et al. in 1989 [100], who demonstrated the effects of daily administration of ∆^9^-THC, a partial CB1R agonist with limited effects on CB2R, on EAE progression in rats. Indeed, the development of EAE was ameliorated, indicating the counteracting effect of ∆^9^-THC in the disease [100]. Subsequently, the effects of the phytocannabinoid ∆^8^-THC (Table 2) on EAE were studied by Wirguin et al. [101]. ∆^8^-THC is an analogue of ∆^9^-THC which binds CB1Rs with high affinity and it is much more stable and less psychotropic than ∆^9^-THC. In this study, ∆^8^-THC significantly ameliorated the clinical manifestations of EAE. Among all the possible mechanisms of action postulated, one involved the inhibition of the prostanoid production by action of an active metabolite of ∆^8^-THC. This active metabolite would explain the necessity of the oral, rather intraperitoneal administration due to the first-pass metabolism in the liver [101].

Further research deepened the study on the role of synthetic and endogenous cannabinoids in CREAE animal model. Baker et al. showed evidence that cannabinoid CBRs agonists, in particular R(+)-WIN 55212 (Table 2), ∆^9^-THC, methanandamide (Table 2) and JWH-133 (Table 2) quantitatively ameliorated both tremor and spasticity in CREAE mice [102]. In order to address these effects to the modulation of the ECS and with the aim to understand which of the CBRs was the most involved, animals were pre-treated with CBRs selective antagonists. The results suggested a role of CB1R to control tremor and an implication of both CBRs in the development of spasticity [102]. In the same work was reported that the endocannabinoid palmitoylethanolamide (PEA) (Table 2) caused a transient inhibition of spasticity [102]. However, more recently it was reported that co-administration of PEA with CBD in EAE was not as active as treatment with each compound alone, indicating that these non-psychoactive cannabinoids could have antagonistic interactions in EAE [103].

New findings indicated that cannabinoids could also target the development of progressive forms of MS, using the TMEV-IDD model of disease [104]. In particular, in TMEV-infected mice, WIN-55212-2 (Table 2), arachidonyl-2-chloroethylamide (ACEA), a selective CB1R agonist (Table 2), and JWH-015, a weak selective CB2R agonist (Table 2), was demonstrated to improve motor function on established neurological symptomatology, to promote the remyelination, and to reduce microglial activation and the number of CD4+ infiltrated T cells [105]. Recent studies focused on the development and study of CB2R selective agonists as the best therapeutic approach thanks to their lack of central side effects usually associated with a CB1R modulation. Recent research aimed at the synthesis of CB2R selective ligands bearing different chemical scaffolds to find new agents for the treatment of MS. First of all, a resorcinol derivative, O-1966 (Table 2), was tested in the chronic EAE model. This compound significantly improved motor function in the chronic EAE model, at concentration of 1 mg kg^−1^. Moreover, O-1966 reduced rolling and adhesion of endogenous leukocytes [106]. 

1,4-dihydro-6-methylindeno[1,2-c]pyrazole derivative, Gp-1a (Table 2), with a four log higher affinity for CB2R than for CB1R, was demonstrated to be able to reduce clinical scores and ameliorate the recovery in EAE mice presenting a long term reduction in demyelination and axonal loss. Two different mechanisms were established since it was able to affect Th1/Th17 differentiation in peripheral immune organs and pathogenic T cell accumulation in the CNS and reduce the expression of chemokine and adhesion molecules in the CNS [107].

Furthermore, in 2015, Fu et al. [108] showed that intrathecal administration of JWH-133 (Table 2), a selective CB2R agonist, in EAE mice, dose-dependently reduced both mechanical and cold hypersensitivity without any signs of ataxia or sedation. The co-administration of JWH-133 with a selective CB2R antagonist dose-dependently attenuated the inhibitory effects of JWH-133. This data suggested that the selective targeting of spinal CB2R reduced signs of neuropathic pain in EAE mice without any side effects [108].

In the same year, Han et al. synthesized new quinoline-2,4(1H,3H)-dione derivatives as CB2R agonists. Among all the synthesized derivatives, compound 21 (Table 2) was the one shown to significantly reduce the clinical scores and symptoms of the mice EAE model, as shown by the remarkably decreased leukocyte infiltration in the spinal cord and demyelination in white matter [109]. 

The following year, chromenopyrazole was identified as a promising scaffold to obtain CBRs ligands [110]. Structural modifications have been studied in order to achieve CB2R selectivity and the structural changes led to the synthesis of chromenoisoxazole derivative PM-226 (Table 2) that was shown to be fully CB2R selective with a high affinity constant. PM-226 was found to dampen neuroinflammation in the TMEV mouse model by reducing microglial activation to levels close to those quantified in the control group [110]. This decrease in the microglia activation lead, as already reported, to a reduction of inflammatory events and an improvement of the neurological status of treated animals [111].

In 2017, Ying Shi et al. reported the identification of new potent and selective indole based CB2R agonists [112]. Compound 57 (Table 2) was selected as a representative analogue to be studied in a mouse EAE model of MS. This compound was significantly shown to alleviate the clinical symptoms and to protect the murine central nervous system from immune damage in EAE mouse model. Further histological examination of spinal cords demonstrated significant reduction in leukocyte infiltration and the extent of demyelination [112]. This study supporting again the efficacy of selective CB2R agonists in animal models of MS.

Very recently, Navarrete et al. [113] provided evidence that VCE-004.8 (Table 2), an aminoquinone derivative of cannabidiol (CBD), is a promising small molecule to modulate relevant MS targets, being a dual PPARγ and CB2 agonist with potent anti-inflammatory activity. VCE-004.8 showed immunomodulatory activity in EAE and TMEV mice models, inhibiting several inflammatory chemokines, chemokines receptors, and cytokines that play a key role in the pathogenesis of MS. In addition, VCE-004.8 inhibited the expression of adhesion molecules such as VCAM and ICAM-1. Also remarkable is the finding that VCE-004.8 strongly induced the expression of the hypoxia-inducible factor (HIF), which can have a beneficial role in MS by modulating the immune response and favoring neuroprotection and axonal regeneration. 

Regarding phytocannabinoids, excluding CBD and ∆^9^-THC, which are discussed in a specific section of this review, the sesquiterpene β-caryophyllene (BCP) (Table 2) is worth mentioning. It is a CB2R selective agonist already reported in literature for its anti-inflammatory and analgesic effects in mouse models of inflammatory and neuropathic pain [114]. In a very recent work, BCP was shown to attenuate disease progression by reducing mechanical hyperalgesia, inflammation, and pain in a mouse EAE model [115]. In order to prove that BCP effects were due to its actions on CB2Rs, BCP was co-administered with a selective CB2R antagonist, which reversed the BCP effects. Altogether, this data suggested that BCP, binding to CB2R, blocks the development and progression of clinical and neurological signs of EAE [115]. 

### 3.2. Inhibitors of Metabolic Enzymes of ECs

Alternative strategies to modulate ECS are focused on blocking the enzymes that degrade the two main endocannabinoid compounds (2-AG and AEA). This is an interesting therapeutic approach, as enhancing ECs levels is expected to preserve the beneficial effects derived from the direct activation of CBRs but limiting potential side effects mostly associated to direct CB1R agonists. It has been widely demonstrated that, in MS patients, there is a significant alteration of the metabolic enzymes mainly of FAAH and of MAGL [116,117].

The effects linked to alterations of the metabolism of endocannabinoids are not completely clear, also because of the different experimental models used and the variations in the recruitment of patients. Different studies in TMEV-IDD mice have assessed that the inhibition of AEA degradation by FAAH determines an improvement of the motor symptoms, with a reduction of inflammatory response and the downregulation of macrophage and of microglial function [118,119]. Webb et al. have demonstrated, in tests carried out on an EAE mouse model, that chronic and long-term inhibition of FAAH, via genetic ablation, produces clinical remission and ameliorates long-term results [120]. Other researchers have shown that high levels of 2-AG can make improvements in the acute phase of MS. Indeed, 2-AG is able to inhibit spasticity when administered at dose of 10 mg kg^−1^ and determines a delayed onset in acute and chronic EAE models when given at a concentration of 100 mg kg^−1^ [121].

The focus of the researchers has been mainly on the MAGL inhibitors, as 2-AG is the main endocannabinoid present in the brain, and it is a full agonist of CB1R and CB2R. However, Scholosburg et al. [122] have shown that increased levels of 2-AG in the brain, due to the chronic MAGL inhibition, provokes a functional antagonism of the cerebral endocannabinoid system [122]. This was evidenced with tolerance to the analgesic effects of acute enzymatic inhibition, cross-tolerance to CB1R agonists, reduction of expression and function of the CB1R, and interruptions in endocannabinoid-dependent synaptic plasticity.

In a recent work, the utility of irreversible MAGL inhibitor, JZL184, (Table 3), in the treatment of MS has been demonstrated [123]. Indeed, the chronic administration of JZL184 reduced the neurological consequences of disease progression in EAE mice. This result is linked to reduce of myelin loss and of inflammation of spinal cord white matter [123]. Furthermore, it was demonstrated that the repeated administration of JZL184 at a dose of 8 mg kg^−1^ did not provoke changes in CB1 receptor expression in the hippocampus. Moreover, there was not tolerance to the anxiolytic and analgesic effects of the MAGL inhibitor [123].

With the aim to increase the potency of JZL184, Brindisi et al. described the synthesis of new β-lactam-based inhibitors reporting compound 4a (Table 3) as very potent hMAGL inhibitor, with high selectivity toward FAAH, other serine hydrolases, and CBRs [124]. This compound in EAE mice showed analgesic effects that were clearly dependent on the increased levels of 2-AG and the subsequent indirect modulation of CBRs. Moreover it exerted a surprising beneficial effect on the progression of the disease, thanks to the CB1R and CB2R mediated action, confirming the hypothesis of a close intersection between the endocannabinoid system and MS. Histological evaluation of myelin showed that, when the EAE mice were treated with compound 4a, myelin-density staining was comparable to that of control animals [124].

As the irreversible MAGL inhibition causes pharmacological tolerance and receptor desensitization, many researchers have focused on development of reversible inhibitors. An interesting example is given by the compound 21 (Table 3) synthesized by Hernández-Torres et al. [125]. This compound showed a submicromolar IC_50_ value for MAGL inhibition and very good selectivity against FAAH, ABDH6, and ABHD12 enzymes, the CB1R and CB2R cannabinoid receptors [125]. In AEA mouse, it was demonstrated its ability to significantly increase the levels of 2-AG in spinal cord, improving clinical symptoms and decreasing tissue damage in the spinal cords. Importantly, catalepsy or other motor impairments that are observed after the administration of irreversible MAGL inhibitors, didn’t occurred.

As reported above, the prolonged inhibition of MAGL enzymes, although effective, provokes negative effects that compromise its biological activity. These effects do not occur by FAAH inhibition. Indeed, it was demonstrated that the prolonged inhibition of FAAH produced no tolerance or no changes in the expression or function of the CB1 receptor [122].

Pryce et al. [126] studied the efficacy of FAAH inhibitors to control the spasticity in Biozzi ABH mice. They demonstrated that potent FAAH inhibitors such as CAY10402 (Table 3) and CAY10400 (Table 3) inhibited spasticity but did not induce any hypothermia, typical of cannabimimetic effects. However, CAY10400 and CAY10402 have poor pharmacokinetics, and therefore, their development as therapeutic drugs is unlikely [126].

A valid alternative is given by compound URB597 (Table 3), which is a potent FAAH inhibitor irreversible, with improved pharmacokinetic profile [126]. Repeated administration of URB597 in a few days compared to the vehicle demonstrated that this compound induced spasticity alleviation immediately after administration, but spasticity was similarly inhibited after four daily doses. However, the study emphasized a benefit because the level of spasticity at baseline after four administrations was lower than baseline before treatment. Moreover, the use of this inhibitor was not associated with CB1R tolerance.

Another way to modulate the endocannabinoid system is to act on AEA reuptake, and many selective inhibitors of cellular reuptake of AEA, but inactive against other enzyme involved in the degradation of this endocannabinoid, have been developed. Ligresti et al. reported very interesting inhibitors of endocannabinoid reuptake [127]. In particular, the most potent compound was O-3246 (Table 3), which has a very high potency as an inhibitor of AEA cellular uptake and a negligible activity as an FAAH inhibitor, CB1R and CB2R ligand, and TRPV1 agonist. This compound was shown to inhibit spasticity in CREAE mice, confirming the potential utility of selective AEA uptake inhibitors as anti-spasticity drugs in MS [127].

Furthermore, it has been found that UCM707 (Table 3), a potent and selective inhibitor of the AEA reuptake [119], was able to improve the motor function in a TMEV-IDD mouse model, and at the histological level, it reduced microglial activation, diminished major histocompatibility complex class II antigen expression, and decreased cellular infiltrates in the spinal cord [119]. These results confirm the role played by the ECS at the level of immunomodulation, and they are in agreement with experiments that describe how the blockade of microglial activation represses the development of the EAE model of MS.

## 4. Conclusions

MS is a neurodegenerative disease affecting millions of people worldwide, yet there is no cure, and the management of symptoms remains a clinical challenge. Recent findings highlight the fact that, in animal models of MS, the modulation of distinct components of ECS (CBRs, degrading enzymes, and AEA transporters) may represent a new and promising therapeutic strategy in the control of symptoms and disease progression in MS. Relief of symptoms in MS by cannabinoids has been reported to be mostly mediated by the activation of CB1R. This amelioration of spasticity can be achieved by the elevation of the endogenous levels of endocannabinoids via inhibition of the AEA transporter as well as by inhibition of the deactivating enzymes of AEA and/or of 2-AG, FAAH, and MAGL, respectively. Moreover, the changes reported for the ECS in different MS models have been associated with adaptive responses for limiting neuronal damage. In particular, the activation of CB1R provides neuroprotection regulating glutamate homeostasis and excitotoxic damage. Furthermore, the protective effects of CB2R activation in microglial cells upon inflammatory-induced CNS damage have been demonstrated in preclinical models of MS. Finally, the enhancing trophic and metabolic support to neurons through astroglial CB1R or CB2R allows us to reduce the excitotoxicity providing neuroprotection. From these data, it is reasonable to assume that the simultaneous modulation of more targets of ECS, combined with conventional therapies, might have more beneficial effects by acting synergistically. This goal might be achieved by multi-target ECS modulators that can directly and indirectly modulate cannabinoid receptor activity by exerting different mechanisms of action, thus offering the possibility of modulating the ECS in a safer and more effective way than single target modulation [128]. However, extensive preclinical studies of this kind molecule are needed, and only positive results from these preclinical studies can lead to a possible clinical study. The multi-target modulators of ECS could be able to control disease progression, and at the same time the symptoms of multiple sclerosis, and might have a translational potential and could represent promising candidates for clinical development.

## Figures and Tables

**Table 1:** Summary of clinical evidence of dronabinol, nabilone, and nabiximols.

	Dronabinol (Synthetic∆^9^-THC)	Nabilone (Synthetic Analogue of ∆^9^-THC)	Nabiximols (∆^9^-THC: Cannabidiol~1:1 (*w/w*))
Structure(s)	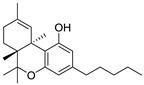	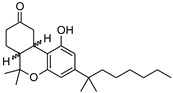	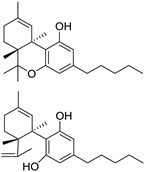
Formulation	Soft gelatin capsules (2.5, 5, 10 mg)	Capsules (0.25, 0.5, 1 mg)	Oro-mucosal spray (27 mg of ∆^9^-THC and 25 mg of cannabidiol/1.0 mL)
Disability and disease progression	No evident changes	No studies	No evident changes
Pain	Positive effects	Positive effects	Mixed findings (mostly positive effects)
Spasticity	Mixed findings	Positive effects	Mixed findings (mostly positive effects)
Bladder function	Mixed findings	Positive effects	Mixed findings
Ataxia and tremor	No evident changes	No studies	No evident changes
Sleep	Mixed findings (mostly positive effects)	No studies	Positive effects
Quality of life	Mixed findings	Mixed findings (moslty positive effects)	Mixed findings
Adverse effects	Mild to moderate. Principally dizziness, euphoria, dry mouth, fatigue and drowsiness.	Moderate sedation, dizziness and moderate weakness in the legs.	Mild to moderate. Principally drowsiness, dizziness, headache, fatigue, impaired balance and disturbance in attention.
Number of studies	10	3	11
Number of reviews	11	5	12
Studies (references)	[45,46,47,48,49,50,51,52,53,54]	[55,56,57]	[58,59,60,61,62,63,64,65,66,67,68]
Reviews (references)	[33,69,70,71,72,73,74,75,76,77,78]	[69,70,72,73,78]	[69,70,72,73,74,75,76,77,78,79,80,81]

**Table 2:** Cannabinoid receptors (CBRs) ligands and their effects shown in different animal models of MS (pertinent references are in parenthesis).

Structure and Name	Origin and Activity	Animal Model and Effects
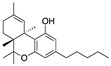 **∆^9^-THC**	Phytocannabinoid CB1R partial agonist	In EAE rats: amelioration of EAE progression [100]. In CREAE mice: amelioration of tremor and spasticity [102].
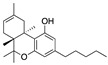 **∆^8^-THC**	Phytocannabinoid CB1R ligand	In EAE rats: amelioration of the clinical manifestations of EAE [101].
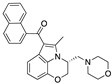 **WIN-55212**	Synthetic cannabinoid CB2R agonist	In CREAE mice: amelioration of tremor and spasticity [102]. In TMEV-infected mice: improvement of motor function on established neurological symptomatology; stimulation of the remyelination; reduction of microglial activation and of the number of CD4+ infiltrated T cells [105].
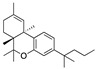 **JWH-133**	Synthetic cannabinoid CB2R agonist	In CREAE mice: amelioration of tremor and spasticity [102]. Intrathecal administration in EAE mice: reduction, dose-dependently, of both mechanical and cold hypersensitivity without any signs of ataxia or sedation [108].
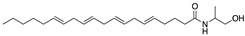 **Methanadamide**	Endocannabinoid CB1R/CB2R agonist	In CREAE mice: amelioration of tremor and spasticity [102].
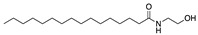 **Palmitoylethanolamide (PEA)**	Endocannabinoid CB1R/CB2R agonist	In CREAE mice: transient inhibition of spasticity [102].
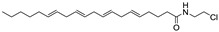 **Arachidonyl-2-chloroethylamide (ACEA)**	Synthetic cannabinoid CB1R agonist	In TMEV-infected mice: improvement of motor function on established neurological symptomatology; stimulation of the remyelination; reduction of microglial activation and of the number of CD4+ infiltrated T cells [105].
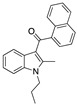 **JWH-015**	Synthetic cannabinoid CB2R agonist	In TMEV-infected mice: improvement of motor function on established neurological symptomatology; stimulation of the remyelination; reduction of microglial activation and of the number of CD4+ infiltrated T cells [105].
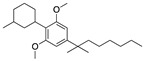 **O-1966**	Synthetic cannabinoid CB2R agonist	In the chronic EAE model: improved motor function; reduction of rolling and adhesion of endogenous leukocytes to pial microvasculature [106].
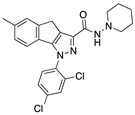 **Gp-1a**	Synthetic cannabinoid CB2R agonist	In EAE mice: reduction of clinical scores; amelioration of the recovery [107].
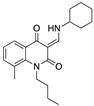 **compound 21**	Synthetic cannabinoid CB2R agonist	In EAE mice: reduction of the clinical scores and symptoms; decrease of leukocyte infiltration in the spinal cord and demyelination in white matter [109].
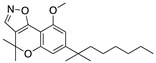 **PM-226**	Synthetic cannabinoid CB2R agonist	In TMEV-infected mice: dampening of neuroinflammation; reduction of microglial activation [110,111].
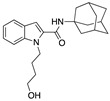 **compound 57**	Synthetic cannabinoid CB2R agonist	In EAE mice: alleviation of the clinical symptoms of EAE; protection of the murine central nervous system from immune damage; reduction of leukocyte infiltration and demyelination [112].
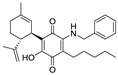 **VCE-004.8**	Synthetic cannabinoid CB2R agonist	In EAE and TMEV mice: immunomodulatory activity; inhibition of inflammatory chemokines, chemokines receptors, and cytokines; inhibition of the expression of adhesion molecules (VCAM and ICAM-1); induction of the expression of the hypoxia-inducible factor (HIF) [113].
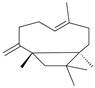 **β-caryophyllene (BCP)**	Phytocannabinoid CB2R agonist	In EAE mice: reduction of mechanical hyperalgesia, inflammation and pain [115].

**Table 3:** Inhibitors of metabolic enzymes of endocannabinoids (ECs) and their effects shown in different animal models of multiple sclerosis (MS) (pertinent references are in parenthesis).

Structure and Name	Activity	Animal Model Effects
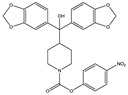 **JZL 184**	Irreversible MAGL inhibitor	In EAE mice: reduction of myelin loss; reduction of inflammation on spinal cord white matter [123]
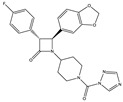 **Compound 4a**	Irreversible MAGL inhibitor	In EAE mice: analgesic effect [124]
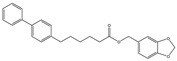 **Compound 21**	Reversible MAGL inhibitor	In EAE mice: decrease of tissue damage in the spinal cords [125]
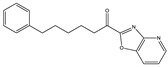 **CAY 10402**	Irreversible FAAH inhibitor	In Biozzi ABH mice: inhibition of spasticity [126]
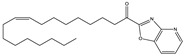 **CAY 10400**	Irreversible FAAH inhibitor	In Biozzi ABH mice: inhibition of spasticity [126]
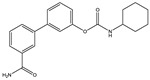 **URB597**	Irreversible FAAH inhibitor	In Biozzi ABH mice: inhibition of spasticity [126]
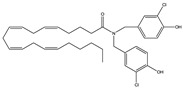 **O-3246**	AEA reuptake inhibitor	In CREAE mice: inhibition of spasticity [127]
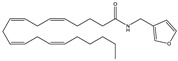 **UCM707**	AEA reuptake inhibitor	In TMEV-IDD mice: improvement of motor function; reduction of microglial activation; decrease of cellular infiltrates in the spinal cord [119]
